# Chimpanzees and bonobos differ in intrinsic motivation for tool use

**DOI:** 10.1038/srep11356

**Published:** 2015-06-16

**Authors:** Kathelijne Koops, Takeshi Furuichi, Chie Hashimoto

**Affiliations:** 1 Anthropological Institute and Museum, University of Zurich, Winterthurerstrasse 190, 8057 Zürich, Switzerland; 2 Department of Archaeology & Anthropology, University of Cambridge, Pembroke Street, CB2 3QG Cambridge, United Kingdom; 3 Primate Research Institute, Kyoto University, Aichi 484-8506, Japan

## Abstract

Tool use in nonhuman apes can help identify the conditions that drove the extraordinary expansion of hominin technology. Chimpanzees and bonobos are our closest living relatives. Whereas chimpanzees are renowned for their tool use, bonobos use few tools and none in foraging. We investigated whether *extrinsic* (ecological and social opportunities) or *intrinsic* (predispositions) differences explain this contrast by comparing chimpanzees at Kalinzu (Uganda) and bonobos at Wamba (DRC). We assessed ecological opportunities based on availability of resources requiring tool use. We examined potential opportunities for social learning in immature apes. Lastly, we investigated predispositions by measuring object manipulation and object play. Extrinsic opportunities did not explain the tool use difference, whereas intrinsic predispositions did. Chimpanzees manipulated and played more with objects than bonobos, despite similar levels of solitary and social play. Selection for increased intrinsic motivation to manipulate objects likely also played an important role in the evolution of hominin tool use.

Chimpanzees (*Pan troglodytes*) and bonobos (*Pan paniscus*) are humans’ closest living relatives and the two sister species shared a common evolutionary history until about one million years ago^1^. Yet, despite their evolutionary closeness, chimpanzees and bonobos differ in a number of important ways. One of the most striking differences is the discrepancy in the reliance on tool use in wild populations of *Pan*. The use of tools by our primate relatives can help us to identify the conditions that drove the extraordinary expansion of hominin technology.

Chimpanzees are renowned for their extensive use of tools in a wide variety of contexts, including feeding, self-maintenance and social contexts^2^. All chimpanzee populations studied in the wild use tools, although the prevalence of tool use varies significantly across groups^3^. The geographic variation in tool use across chimpanzee populations is most parsimoniously explained in terms of culture^3^,^4^. Chimpanzee cultural variants include nut cracking, termite fishing and ant dipping.

Bonobos, on the other hand, use surprisingly few tools in the wild and no tool use in feeding has been observed^5^,^6^,^7^. The use of tools by wild bonobos mainly concerns social contexts, comfort and protection from the rain. In contrast, most chimpanzee tool use occurs in a foraging context, especially in extracting difficult to obtain foods^8^. Previously, the absence of bonobo feeding-tool use has been proposed to result from year-round high food availability^5^,^9^, limiting the necessity for tool use. However, a recent comparison between chimpanzee and bonobo habitat characteristics and fluctuation of fruit production failed to support this hypothesis^8^.

In chimpanzees, extrinsic factors, in terms of ecological opportunities for tool use, play an important role in explaining *within* species differences in tool use^10^,^11^. Chimpanzees and bonobos are allopatric and thus inhabit different environments, which raises the possibility that ecological differences may also play an important role in explaining *between* species tool use differences. Moreover, similar levels of tool use in captive chimpanzees and bonobos^12^, kept under similar conditions, suggest that the species difference in tool use may be due to differences in extrinsic (ecological or social) opportunities for tool use in the wild. Alternatively, chimpanzees and bonobos may differ intrinsically (i.e. not in response to external stimuli), in terms of their predisposition, or intrinsic motivation, for tool use.

We investigated whether *extrinsic* (ecological and social opportunities) or *intrinsic* (predispositions) differences explain the tool use difference in a comparative study of chimpanzees (*P. t. schweinfurthii*) in the Kalinzu Forest (Uganda) and bonobos at Wamba (Democratic Republic of Congo). Chimpanzees at Kalinzu are known to use sticks to harvest army ants^13^, whereas bonobos at Wamba use tools only in a non-feeding context, e.g. twig as a rain hat or branch drag in display^8^.

We assessed the ecological opportunities for tool use based on the availability of insects requiring tools for harvesting (i.e. army ants, *Macrotermes*), nut trees and stones. Bonobos are exceptionally gregarious, with party sizes exceeding those in chimpanzees^14^. We investigated whether or not gregariousness reflects close-range social learning opportunities^15^,^16^. We assessed the potential opportunities for social learning based on focal observations of immature chimpanzees and bonobos. We measured time (%) spent in close proximity to the mother and to other individuals in feeding and non-feeding contexts, the mean number of individuals in close proximity, and the total number of social partners. Lastly, we investigated the predisposition for tool use by measuring tool use precursors^17^ as observable proxies of tool use tendencies, namely object manipulation and object play.

## Results

### Ecological opportunities

**Figure 1:**
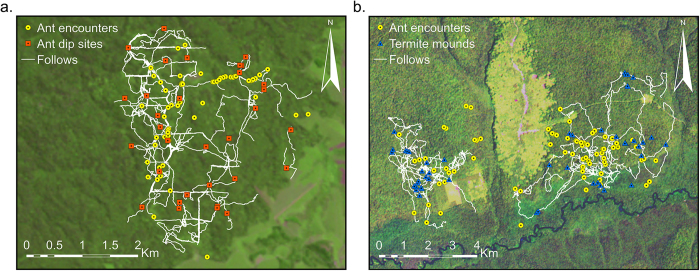
Distribution of army ants, *Macrotermes* mounds and ant dipping sites in relation to chimpanzee and bonobo follows at Kalinzu and Wamba (maps created using ESRI ArcMap 10.1 and Landsat images) (**a**) Chimpanzee follows (white lines) with army ant encounters (yellow dots) and ant dip sites (red squares) at Kalinzu. (**b**) Bonobo follows (white lines) with army ant encounters (yellow dots) and *Macrotermes* termite mounds (blue triangles) at Wamba for E1 group (right) and P group (left).

We recorded two species of army ants at Kalinzu (*D. terrificus, D. wilverthi*) and four species at Wamba (*D. opacus, D. rubelus, D. sjoedstedti, D. wilverthi*). Mean ant density at Kalinzu was 0.50 trails/km, which was not significantly different from 0.80 trails/km at Wamba (T-test: t = −1.8, df = 15, *P* = 0.087). *Macrotermes* mounds were absent at Kalinzu and one species (*M. muelleri*) at 0.2 mounds/ha was recorded at Wamba. Nut trees at Kalinzu consisted of *Parinari excelsa* at 0.39 trees/ha. At Wamba, three nut tree species were recorded: *Elaeis guineensis* (10 trees/ha)*, Parinari excelsa* (1.1 trees/ha) and *Panda oleosa* (0.2 trees/ha). Overall nut tree densities did not differ significantly between Wamba and Kalinzu (T-test: t = −2.5, df = 15, *P* = 0.065). Stones were absent below nut trees at both sites. The spatial distribution of army ants (*n* = 189) at Kalinzu overlapped with the chimpanzees’ home range (Fig. 1a). Moreover, ant-dipping sites (*n* = 35) were recorded across the chimpanzees’ range (Fig. 1a). The distribution of army ant encounters (*n* = 55) and *Macrotermes* mounds (*n* = 48) at Wamba also overlapped with the two bonobo home ranges (Fig. 1b).

### Social opportunities

We assessed the social opportunities for learning available to immature chimpanzees and bonobos by comparing the proportion of time spent in close proximity to their mothers, as well as to other individuals. Bonobos spent more time <2 m from their mother compared to chimpanzees (ANCOVA: Species: F_1, 27_ = 6.53, *P* = 0.017, Age: F_1, 27_ = 127.6, *P* < 0.0001), when controlling for age. There was no difference in time spent <2 m from other individuals in a non-feeding context (Species: F_1, 26_ = 1.31, *P* = 0.264, Age: F_1, 26_ = 2.87, *P* = 0.102). However, in a feeding context, bonobos spent more time <2 m from individuals other than their mother compared to chimpanzees (Species: F_1, 18_ = 13.3, *P* = 0.002, Age: F_1, 27_ = 0.07, *P* = 0.795). Next, we compared the mean number of individuals observed within a 2-m radius. Bonobos had more individuals in close proximity than chimpanzees (Species: F_1, 26_ = 5.86, *P* = 0.023, Age: F_1, 26_ = 0.002, *P* = 0.968). Lastly, we compared the number of social partners. Young bonobos had more social partners than young chimpanzees (Species: F_1, 26_ = 50.5, *P* < 0.0001, Age: F_1, 26_ = 0.576, *P* = 0.455), as well as more unrelated social partners (Species: F_1, 26_ = 39.1, *P* < 0.0001, Age: F_1, 26_ = 0.118, *P* = 0.734).

### Predispositions

**Figure 2:**
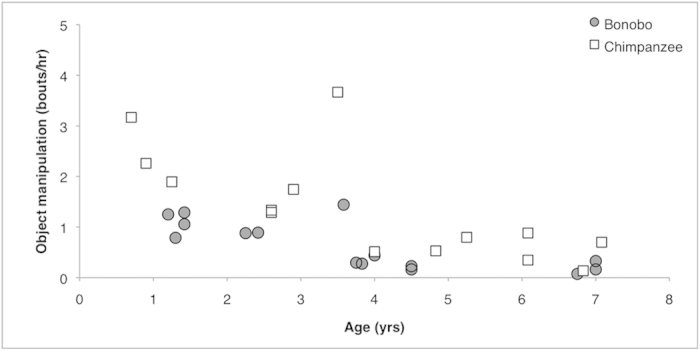
Object manipulation rates according to age for bonobos and chimpanzees Relationship between each subject’s age and its object manipulation rates (bouts/hour) for bonobos (grey circles) and chimpanzees (white squares).

We assessed the predispositions for tool use by comparing object manipulation and object play. The object types manipulated by both species were: leaves, woody vegetation, fruits and moss. In addition, one immature chimpanzee manipulated inedible seeds and one bonobo played with a bird’s nest. First, we compared object manipulation rates between feeding and resting contexts. None of the feeding contexts involved tool use. Object manipulation rates were higher in a resting compared to a feeding context in both chimpanzees (Wilcoxon Signed Rank test: *z* = 2.5, *P* = 0.013) and bonobos (*z* = 2.5, *P* = 0.013). Moreover, object manipulation rates were higher in chimpanzees than in bonobos, both for detached objects (Species: F_1, 27_ = 15.2, *P* = 0.001, Age: F_1, 27_ = 28.6, *P* < 0.0001) and for all objects (detached and attached), excluding nest building (Species: F_1, 27_ = 20.1, *P* < 0.0001, Age: F_1, 27_ = 41.6, *P*   < 0.0001, Fig. 2).

**Figure 3:**
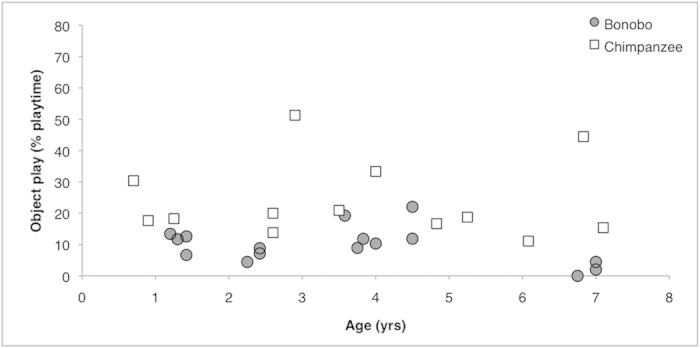
Object play according to age for bonobos and chimpanzees Relationship between each subject’s age and object play (% playtime) for bonobos (grey circles) and chimpanzees (white squares).

In addition, we compared object play between young chimpanzees and bonobos. The proportion of time in object play was higher for chimpanzees than for bonobos (Species: F_1, 26_ = 5.6, *P* = 0.026, Age: F_1, 26_ = 30.6, *P* < 0.0001). This result increased in significance when comparing the proportion of total *playtime* in object play (Species: F_1, 26_ = 20.4, *P* < 0.0001, Age: F_1, 26_ = 2.5, *P* = 0.130, Fig. 3). We also compared the proportion of time in solitary and social play. There was no species difference in the proportion of time playing alone (F_1, 25_ = 0.543, *P* = 0.468, Age: F_1, 25_ = 105.8, *P* < 0.0001), or together (F_1, 26_ = 1.06, *P* = 0.312, Age: F_1, 26_ = 3.42, *P* = 0.076). Lastly, we compared different play types (solitary and social, with and without objects). The only difference was found in the amount of solitary play *with* objects: chimpanzees played more with objects on their own than bonobos (Mann-Whitney U test: *z* = 3.3, *P* = 0.001).

## Discussion

We investigated whether extrinsic or intrinsic differences could explain the remarkably difference in the reliance on tools between wild chimpanzees and bonobos. Our findings showed that ecological and social opportunities did not explain the species difference in tool use. Predispositions, however, did.

We recorded more army ant species at Wamba than at Kalinzu, but army ant densities did not differ significantly. No tools were used to capture army ants by bonobos at Wamba. Tool-assisted army ant predation has been reported for chimpanzee sites with ant densities much lower than Wamba (0.80 ants/km). For example, army ant density is 0.08 ants/km at Seringbara, Guinea, and chimpanzees here do use tools to harvest army ants^10^,^18^. Hence, bonobos at Wamba had sufficient ecological opportunities to dip for army ants.

*Macrotermes* mounds were absent at Kalinzu. The species recorded at Wamba, *M. muelleri*, is harvested with tools by chimpanzees at Goualougo, Republic of Congo, and at La Belgique, Cameroon^19^. Mound density at Wamba was 0.2 mounds/ha, which is comparable to densities at Gombe (0.7 mounds/ha) and Mahale-Belinge (0.1 mounds/ha), two Tanzanian sites where chimpanzees termite fish^20^. Hence, Wamba bonobos had ample ecological opportunities to fish for termites. We did not asses tool availability for ant dipping and termite fishing, since raw materials for tools have been shown to be abundant in the forest of the Congo Basin^21^.

At neither Wamba nor Kalinzu did the apes crack nuts using tools. Nut tree availability was more varied in Wamba with four species compared to one at Kalinzu. However, *Coula edulis*, a high value nut and catalyst for cracking of other species^10^, was absent at both sites. Moreover, neither at Wamba nor at Kalinzu did we record stones under nut trees. Chimpanzees crack nuts with wooden hammers in Taï, Ivory Coast^22^, but this concerns the softer *C. edulis* nuts. Hence, ecological opportunities for nut cracking were present at both sites, albeit limited.

Bonobos spent more time in close proximity to their mothers than chimpanzees. There was no species difference in the amount of time spent close to other individuals in a non-feeding context, suggesting that party size differences between the two species did not automatically result in more time in close proximity to others. However, in a feeding context, bonobos did spend more time in close proximity to other individuals than chimpanzees. These findings confirm higher levels of feeding tolerance in bonobos compared to chimpanzees previously reported for captive apes^23^. In sum, young bonobos had more potential opportunities to learn from their mothers, as well as from others when it comes to feeding skills.

In addition, young bonobos had more individuals in close proximity, as well as more social partners than chimpanzees. Recent findings on captive apes reported bonobos as less socially tolerant than chimpanzees and gorillas, since bonobos had fewer neighbours present during tool use^24^. However, fewer neighbours during a tool use task could reflect a lack of interest, rather than a lack of tolerance. Our findings suggest that higher levels of gregariousness and tolerance in bonobos lead to more close-range social learning opportunities and increased access to a variety of social partners from whom to learn.

Object manipulation rates were higher in a resting as compared to a feeding context in both species. Objects manipulated by immature chimpanzees did not include tools used by adults. In fact, immature chimpanzees often manipulated leaves; a material not used by adult Kalinzu chimpanzees in foraging tool use. This suggests that object manipulation by immatures was almost certainly not socially induced, and thus not simply a result of young individuals emulating adult feeding tool use. The different object types targeted by immature chimpanzees and bonobos deserve further investigation (Koops *et al.* under review).

Chimpanzees showed higher rates of object manipulation than bonobos. This difference was apparent already in the youngest individuals observed (<1 yr old), who have experienced relatively few potential social-learning opportunities. Hence, this further suggests an intrinsic, rather than a directly socially facilitated, difference. Our findings provide the first evidence for a species difference in object-orientedness, which likely reflects a difference in the predisposition for tool use in *Pan*.

We showed that young chimpanzees and bonobos also differed with regard to object play. Chimpanzees played more with objects than bonobos. This finding was not a consequence of differences in solitary or social play. The species difference in object play was caused by a difference in solitary play *with* objects, which was observed more in chimpanzees than in bonobos. These results highlight the important role of play in fine-tuning adult behaviours^25^.

Chimpanzees engaged more in object manipulation and object play than bonobos, consistent with a species difference in the intrinsic motivation for tool use. A similar difference in object manipulation was found between the tool-using New Caledonian crows, *Corvus moneduloides*, and the non-tool using common ravens, *Corvus corax*^26^. In both corvids and apes, the predisposition to manipulate objects likely results from a general increase in object-orientedness, rather than a difference in general cognitive capacity.

The question is whether bonobos lost the predisposition for tool use and if so, *why*? A recent study on captive apes using eye-tracking techniques found a species difference in attention bias. Bonobos pay more attention to social cues, whereas chimpanzees pay more attention to the action target object (Kano *et al.* under review). Together with our findings this suggests a trade-off between tool use motivation and social attention. The next step will be to assess predispositions across different populations of chimpanzees and bonobos. If there exists a connection between predispositions and the presence of tool use in chimpanzee populations, this would provide evidence for culture-gene co-evolution.

To conclude, the tool use dichotomy in *Pan* is driven by *intrinsic* differences in terms of the motivation to interact with objects. Given their close evolutionary relationship with humans, insights into the tool use difference in *Pan* can help us identify the conditions that drove the evolution of human technology. An intrinsic motivation to manipulate objects was likely also selected for in the hominin lineage.

## Methods

### Study sites and subjects

The Kalinzu Forest Reserve is located in western Uganda (30° 07’ E, 0° 17’ S^27^;). The forest is classified as medium-altitude moist evergreen forest^28^,^29^. We collected data on immature members (0.7–7.1 yrs) of the main study community (M-group), which consisted of 97 individuals (19 adult males, 29 adult females). Data collection took place during 3 months from December 2012 - March 2013. The Wamba study site is located in the northern sector of the Luo Scientific Reserve in the Democratic Republic of Congo (research camp: 0° 11’ 08” N, 22° 37’ 58” E^30^;). The habitat comprises primary, old secondary, young secondary and swamp forest^31^. There are two neighbouring study groups: E1-group (31 individuals: 7 adult males, 9 adult females) and P-group (28 individuals: 5 adult males, 7 adult females). We collected data on immature members (1.2–6.8 yrs) of both communities. Data collection took place during 3 months from May - August 2013.

### Data collection

To assess the ecological opportunities for tool use, we measured the availability of army ants (*Dorylus* spp.), termites (*Macrotermes*), nut trees and stones at both study sites. To obtain insect density estimates, we recorded army ants and *Macrotermes* mounds along pre-existing transects in two consecutive months. Total transect length at Wamba was 22.6 km (*n* = 5, mean = 4.5, SD = 0.69, range = 3.6–5.0). Total transect length at Kalinzu was 54 km (*n* = 12, mean = 4.5, SD = 0.61, range = 3.3–5.0). To assess insect distribution in relation to ape distribution, we recorded insect encounters *all occurrences*^10^,^18^. In addition, we recorded all fresh ant-dipping sites with tools present. We recorded the GPS coordinates when encountering army ants, ant-dipping sites or occupied *Macrotermes* mounds. We examined whether or not the apes were exposed to army ants and *Macrotermes* mounds by comparing ape ranging data (see *Behavioural data collection*) to GPS locations of insect encounters. We took samples of army ants and termites for species identification. In addition, we measured the availability of nut trees (i.e. *Elaeis guineensis, Coula edulis, Panda oleosa, Parinari excelsa*) at both sites along the pre-existing transects. We recorded all nut trees (DBH ≥ 10 cm) within 5 m of the transect line. Lastly, we recorded availability of stones in a 5-m radius below nut trees.

Behavioural data collection involved focal animal sampling^32^. We collected data on 14 chimpanzees (7 males, 7 females) in M-group and 16 bonobos (7 males, 9 females) in E1-group (*n* = 9) and P-group (*n* = 7) between 0.7–7.1 years old. Supplementary Tables 1 and 2 provide further detailed information on the focal individuals. At Kalinzu, we collected 109.3 observation hours (mean observation time per individual = 7.9 hours). At Wamba, we collected 210.6 observation hours (mean observation time per individual = 13.2 hours). All focal individuals were followed on at least three different days and observation times were balanced for time of day. Focal individuals were followed for 30–120 minutes per follow (mean duration: bonobos: 81.7 minutes, chimpanzees: 75.6 minutes). Every two minutes, we recorded the focal animal’s general activity (i.e. feed, rest, move, play, groom) and location (i.e. terrestrial, arboreal). Play was divided into *social play* (i.e. play with one or more other individuals) and *solitary play* (i.e. play alone). We also recorded the general activity of the focal individual’s mother and of the majority (>50%) of the party members, which provided the behavioural context (i.e. foraging, resting, travelling) for further analyses. To assess the potential social opportunities for tool use, we recorded the identity of all individuals present within a 2-m radius from the focal individual every two minutes. In addition, for all social interactions involving the focal individual we noted the identity of the social partner(s) in order to obtain a measure of the total number of social partners for each focal individual. To assess the predispositions for tool use we used *object play* and *object manipulation* as observable proxies of tool use tendencies. Object play was defined as all playful manipulation of objects with no apparent goal, including repetitive movements. In addition to scoring object play (i.e. 2-minute activity score) we recorded all object manipulation bouts during focal follows. An object manipulation bout was defined as continuous manipulation of the same object with hands, feet or mouth. A new bout was scored when the object(s) changed or when there was a 2 min break in object manipulation. For all object manipulation bouts, we noted type of manipulation, type of object, and whether or not the object was detached. Ranging data were collected continuously during focal follows with a GPS unit (Garmin Map 60CSX).

### Statistical analyses

We tested data for normality using a normal probability plot and a Kolmogorov-Smirnov test^33^. All analyses were two-tailed and significance levels were set at 0.05. We performed statistical tests in IBM SPSS version 21.0. To compare the ecological opportunities for tool use between the two sites we analysed the insect and nut tree densities. We compared mean army ant densities and nut tree densities using Mann-Whitney U Tests.

To compare the opportunities for social learning between chimpanzees and bonobos we analyzed the time (% observation time) focal individuals spent at a distance of <2 m from their mother and from other individuals in both a non-feeding and a feeding context. We conducted analyses of covariance (ANCOVA) with the factor species (chimpanzee *versus* bonobo), % time <2 m as the dependent variable, and age as the covariate. For the analyses in the feeding context we did a logarithmic transformation to obtain a normal distribution, and we excluded individuals with <50 feeding data points (*n* = 6 chimpanzees, *n* = 3 bonobos). We also compared the mean number of individuals observed at <2 m from focal individuals using an ANCOVA with age as covariate. Lastly, we compared the total number of social partners between immature chimpanzees and bonobos by using an ANCOVA with age as covariate. We had to exclude one chimpanzee (Ua) from the above analyses, since we had only one data point in which an individual other than the mother was at <2 m.

To compare the predispositions for tool use between chimpanzees and bonobos we analyzed object play and object manipulation as observable proxies. First, we compared object manipulation rates (bouts/hour) between feeding and resting context for both species with a Wilcoxon Signed Rank test. Next, we conducted analyses of covariance with the factor species (chimpanzee *versus* bonobo), object manipulation rate as the dependent variable, and age as the covariate. For this analysis we did a logarithmic transformation to obtain a normal distribution and homogeneity of variances. We also compared the amount of object play (% time), solitary play (% time) and social play (% time) between the two species using ANCOVAs with age as covariate. We did a square root transformation for object play and a Logit transformation for solitary play to obtain normal distributions and homogeneity of variances. Lastly, we compared play types (solitary play *with* and *without* objects and social play *with* and *without* objects) for both chimpanzees and bonobos by using Mann-Whitney U tests. We had to exclude one chimpanzee (Ua) from the above analyses, since she did not play with other individuals. We excluded one bonobo (Hideko) from the analysis of solitary play, since she did not play alone.

## Additional Information

**How to cite this article**: Koops, K. *et al.* Chimpanzees and bonobos differ in intrinsic motivation for tool use. *Sci. Rep.* **5**, 11356; doi: 10.1038/srep11356 (2015).

## Supplementary Material

Supplementary Information
