# Supplementary material for: Chimpanzees and bonobos differ in intrinsic motivation for tool use
Source: Sci Rep. 2015 Jun 16;5:11356. doi: 10.1038/srep11356 (PMC4468814; doi:10.1038/srep11356)
Supplement: Supplementary Information [file srep11356-s1.pdf]

SUPPLEMENTARY INFORMATION

Chimpanzees and bonobos differ in intrinsic motivation for tool use

Kathelijne Koops[a,b*], Takeshi Furuichi[c] and Chie Hashimoto[c]

[a]*Anthropological Institute and Museum, University of Zurich, Winterthurerstrasse 190, 8057 Zürich, Switzerland*

[b]*Department of Archaeology & Anthropology, University of Cambridge, Pembroke Street, CB2 3QG Cambridge, United Kingdom*

[c]*Primate Research Institute, Kyoto University, Aichi 484-8506, Japan*

***Corresponding Author:**

Kathelijne Koops

Anthropological Institute & Museum

University of Zurich

Winterthurerstrasse 190

8057 Zürich

Switzerland

Email: kathelijne.koops@uzh.ch

Phone: +41 (0) 78 874 35 02; +44 (0) 77 943 31 849

Supplementary Table 1. Focal individuals (name, sex, age), name of mother and total observation time per individual at Kalinzu

Name	*Sex*	*Age (yrs)*	*Mother*	*Observation time (hrs)*
Mugisha	Male	0.7	Mitsu	4.7
Hayato	Male	0.9	Haro	9.7
Eta	Male	1.3	Esunzu	9.5
Ayu	Female	2.6	Asa	5.4
Picasso	Male	2.6	Pinka	9.0
Iyo	Female	2.9	Ida	9.2
Max	Male	3.5	Mami	6.0
Gale	Female	4.0	Gai	9.7
Haruka	Female	4.8	Haro	9.4
Taro	Male	~5.3	Tae	10.0
Milk	Male	6.1	Mitsu	3.4
Ua	Female	6.1	Ume	5.8
Piriko	Female	~6.8	Pinka	7.4
Iku	Female	7.1	Ida	10.0

Supplementary Table 2. Focal individuals (name, sex, age), name of mother and total observation time per individual at Wamba

Name	*Group*	*Sex*	*Age (yrs)*	*Mother*	*Observation time (hrs)*
Kale	P	Male	1.2	Kabo	12.0
Isao	P	Male	1.3	Ichi	12.7
Hideo	P	Male	2.3	Hide	12.5
Pipi	P	Female	4.5	Pao	12.3
Ichiko	P	Female	4.5	Ichi	13.2
Hideko	P	Female	7.0	Hide	12.3
Kaboko	P	Female	7.0	Kabo	12.2
Seko	E1	Male	1.4	Sala	13.2
Jolie	E1	Female	1.4	Jacky	14.2
Fua	E1	Female	2.4	Fuku	13.5
Otoko	E1	Female	2.4	Otomi	13.5
Yume	E1	Female	3.6	Yuke	13.9
Hachiro	E1	Male	3.8	Hoshi	13.5
Kyota	E1	Male	3.8	Kiku	14.5
Natsuko	E1	Female	4.0	Nao	13.6
Joe	E1	Male	6.8	Jacky	13.5
